# Rare cancer survivorship research funding at the National Institutes of Health (NIH), 2017 to 2023

**DOI:** 10.1007/s10552-025-01959-8

**Published:** 2025-01-21

**Authors:** Lisa Gallicchio, Michelle Mollica, Gina Tesauro, Michelle Doose, Jennifer L. Guida, Molly E. Maher, Emily Tonorezos

**Affiliations:** 1 Epidemiology and Genomics Research Program, Division of Cancer Control and Population Sciences, National Cancer Institute, Bethesda, MD USA; 2 Office of Cancer Survivorship, Division of Cancer Control and Population Sciences, National Cancer Institute, Bethesda, MD USA; 3 Behavioral Research Program, Division of Cancer Control and Population Sciences, National Cancer Institute, Bethesda, MD USA; 4 Healthcare Delivery Research Program, Division of Cancer Control and Population Sciences, National Cancer Institute, Bethesda, MD USA; 5 National Cancer Institute, 9609 Medical Center Drive, Rockville, MD 20850 USA

**Keywords:** National Institutes of Health, Rare cancers, Research funding, Survivorship

## Abstract

**Purpose:**

Rare cancers are defined as those for which there are less than 15 cases per 100,000 in the population annually. While much progress in detection and treatment has been made over the past decade for many rare cancers, less progress has been made in understanding survivorship needs. The objective of this study was to characterize the National Institutes of Health (NIH) cancer survivorship grant portfolio focused on rare cancers and to identify gaps specific to this area of science.

**Methods:**

Newly awarded grants focused on rare cancers in the NIH cancer survivorship research portfolio from Fiscal Year (FY) 2017 to FY2023 were identified. Grant characteristics were abstracted and described. In addition, the number of grants for each rare cancer type was mapped to current Surveillance, Epidemiology, and End Results program incidence and relative survival rates.

**Results:**

A total of 93 survivorship grants focused on one or multiple rare cancer types were funded from FY2017 to FY2023. Approximately 85% of these grants investigated one of four cancer types: leukemia, head & neck, ovarian and brain. Few grants focused on other rare cancer types, such as multiple myeloma (n = 5), testicular cancer (n = 3), rectal cancer (n = 1), thyroid cancer (n = 1), and cervical cancer (n = 0). About half of the grants (50.5%) were observational studies; 34.4% focused explicitly on pediatric cancer survivors.

**Conclusions:**

Survivorship research for many rare cancer types is limited. This paucity of research is a barrier to the identification of survivorship needs and the development of interventions to address these needs.

**Supplementary Information:**

The online version contains supplementary material available at 10.1007/s10552-025-01959-8.

## Introduction

A substantial amount of progress has been made over the last decade in the United States (US) in detecting and treating many rare cancers [1, 2], defined by the National Cancer Institute (NCI) as those for which there are less than 15 cases per 100,000 in the population annually [3]. This progress has resulted in increased survival [4], such that many individuals diagnosed with rare cancers are now living longer, and the goals of their care have changed from cancer cure or palliation at the end of life to addressing survivorship needs. Yet, because of the small number of cases diagnosed relative to the more common cancers, conducting research to understand and address survivorship needs among individuals with rare cancers is challenging. Thus, for many rare cancers, there is a paucity of data on which to guide survivorship care—from diagnosis, through treatment, and to surveillance and management of the possible adverse effects of the cancer diagnosis and its treatment. This lack of research and evidence-based standard of care can further compound health disparities due to other factors that may be associated with the diagnosis of a rare cancer, such as race and ethnicity [5].

Recently, a description of the overall National Institutes of Health (NIH) cancer survivorship grant portfolio from Fiscal Year (FY) 2017–2021 was published [6]; in this publication, rare cancer survivorship was identified as an area in which there are gaps in the cancer survivorship research grant portfolio. The objectives of this analysis were to further characterize the NIH survivorship portfolio focused on rare cancers and to identify gaps specific to the rare cancer survivorship portfolio.

## Methods

This portfolio analysis used the NIH cancer survivorship research portfolio of newly funded grants from FY2017 to FY2023. The detailed search strategy and definitions used to identify this portfolio have been previously described [6]. In brief, NIH research project grants (R series) and cooperative agreements (U series) were identified using a text mining algorithm of words from the NIH Research, Condition, and Disease Categorization (RCDC) thesaurus with survivorship-relevant terms. The title, abstract, and specific aims of the grants were reviewed to determine inclusion. Subsequently, general grant characteristics (e.g. NIH Institute, funding mechanism, notice of funding opportunity (NOFO)) were extracted and grant characteristics (e.g. study design, cancer type, primary focus, outcomes) were coded. Each grant was assessed independently by two co-authors for inclusion into the portfolio and, if included, each grant was separately coded by two co-authors. Disagreements were resolved with discussion.

This analysis included only grants in the NIH cancer survivorship research portfolio focused on rare cancer types (e.g., those with age-adjusted incidence rates less than 15 per 100,000 annually). Incidence rates utilized to identify rare cancers were from the most recent Surveillance, Epidemiology, and End Results (SEER) Program data submission (November 2022) [7]. Only grants with study samples comprised entirely of survivors diagnosed with a rare cancer were eligible for inclusion (i.e., if a grant included survivors with both rare and common cancer types, the grant was excluded). Of the 714 newly funded grants in the overall NIH cancer survivorship research portfolio, 93 grants focused on rare cancers and were included in the analytic dataset.

Descriptive statistics were used to report on the numbers and characteristics of grants funded. To understand the landscape of rare cancer grant funding in comparison to the most up-to-date SEER age-adjusted incidence rates and 1- and 5-year relative survival rates, quadrant charts were created utilizing the most recent SEER statistics (November 2022 submission) [7, 8]. Two diagnoses were excluded from the SEER analysis because the data that were extracted from the grants for cancer type did not match a SEER cancer type category. Head and neck cancers (e.g. oral cavity, larynx) were almost always combined into one group in the grants and thus could not be compared to SEER statistics. In addition, neuroendocrine tumors are not captured as a SEER site, and therefore, could not be evaluated. Analyses were conducted using SAS version 9.4 (SAS Institute, Cary, NC) and Microsoft Excel.

## Results

### Rare cancer survivorship portfolio

**Fig. 1:**
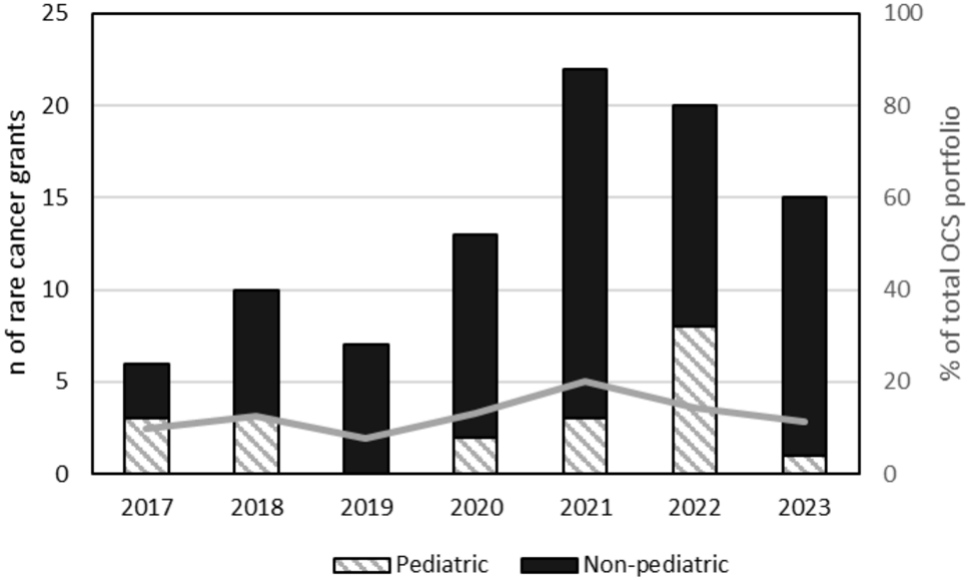
Newly funded cancer survivorship grants focused on rare cancers, by fiscal year

A total of 93 survivorship grants focused on one or multiple rare cancer types were funded from FY2017 to FY2023. In general, the number of grants funded increased over time, from 6 in FY2017 to 15 in FY2023, with a peak of 22 in FY2021 (Fig. 1). In addition, the percentage of grants in the overall NIH cancer survivorship portfolio focused on rare cancers increased over time, from 9.7% in FY2017 to 11.2% in FY2023.

The majority of the rare cancer survivorship grants were funded by the NCI (80.6%), with six other NIH Institutes funding at least one grant from FY2017 to FY2023 (Table 1). More than one third of the grants used the R01 mechanism (40.9%) while approximately one quarter (24.7%) were funded utilizing the R21 mechanism. Grants were most often funded through parent NOFOs (49.5%); 15.0% were funded through Requests for Applications (RFAs) (which have set-aside funds). Approximately 41% of the grants were multiple principal investigator (MPI) grants, and 22.9% of funded grants were awarded to early-stage investigators (ESI).

**Table 1:** Grant characteristics (n = 93)

Characteristic	n	%
*Primary funding institute*		
National Cancer Institute (NCI)	75	80.6
National Heart, Lung, and Blood Institute (NHLBI)	5	5.4
National Institute on Aging (NIA)	4	4.3
National Institute of Dental and Craniofacial Research (NIDCR)	4	4.3
National Institute of Nursing Research (NINR)	3	3.2
National Institute of Child Health and Human Development (NICHD)	1	1.1
National Institute of Neurological Disorders and Stroke (NINDS)	1	1.1
*Grant mechanism*		
R01	38	40.9
R21	23	24.7
R03	8	8.6
U01	5	5.4
R41/R42 (STTR)	1	1.1
R43/R44 (SBIR)	5	5.4
R37	5	5.4
UG3, UH3	3	3.2
R61	2	2.1
R15	1	1.1
R34	1	1.1
R00	1	1.1
*Notice of Funding Opportunity (NOFO) type*		
Request for Applications (RFA)	14	15.0
Targeted (not RFA)	33	35.5
Parent	46	49.5
*Early-stage investigator (ESI)*^a^		
Yes	11	22.9
No	37	77.1
N/A	45	
*Multiple principal investigator*		
Yes	38	40.9
No	55	59.1

^a^ESI status not coded by NIH for RFA-funded grants, as well as the following grant mechanisms: R00, R03, R15, R21, R34, R41, R42, R43, R44, R61 (and others); percentage calculated among those grants for which ESI status is coded

**Fig. 2:**
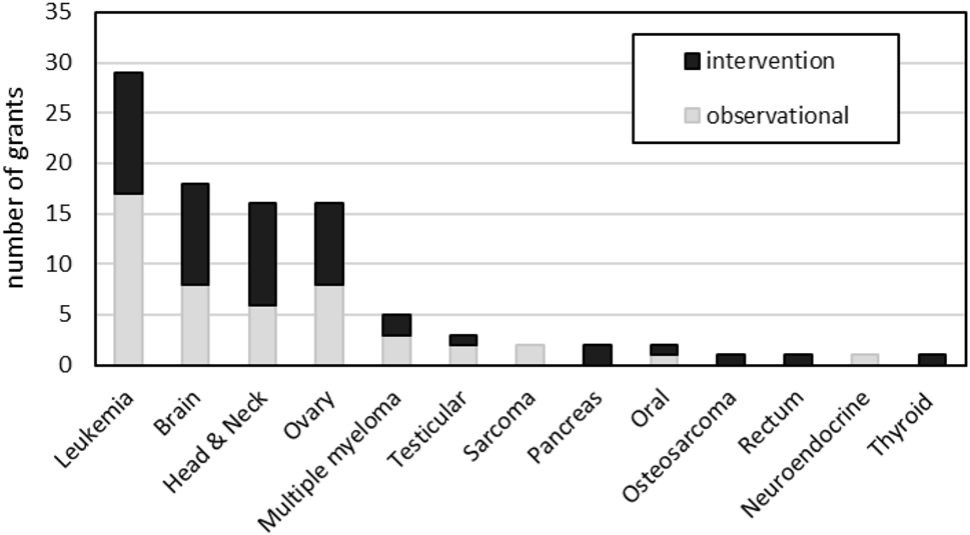
Number of grants by cancer type and study design, fiscal years 2017 to 2023. A grant may be included in multiple cancer type categories

Of the 93 grants in the portfolio, six (6.5%) included more than one rare cancer type. Leukemias (ALL, AML, or CML) were the most common rare cancer type of focus (31.2%); with head and neck, brain, and ovarian cancers each being the focus of approximately one-fifth of the grants in the portfolio (Fig. 2). Most often, study samples were not restricted to a single sex (84.9%) (Table 2), and when there was a single sex, it was because of the cancer type (e.g., ovarian; testicular). There was an explicit focus on pediatric cancer survivors (i.e., age at the time of diagnosis and study enrollment was less than 21 years of age) in a third of the grants (34.4%); 9.7% of grants focused on racial/ethnic minority groups and 9.7% included caregivers. Few grants focused on older adults (6.5%), long-term survivors (5.4%), AYA survivors (7.5%), or adult survivors of pediatric cancers (i.e., cancer was diagnosed at less than 21 years of age, but age of study enrollment was 21 years of age or older) (3.2%).

**Table 2:** Population and study characteristics of NIH rare cancer survivorship grant portfolio (n = 93)

Characteristic	n	%
*Sex*		
Both	79	84.9
Female only	12	12.9
Male only	2	2.2
*Special population of focus*		
Adult survivors of pediatric cancer (diagnosed at < 21 years of age, currently ≥ 18 years of age)	3	3.2
Pediatric survivors (< 21 years of age at the time of study)	32	34.4
Rural cancer survivors	1	1.1
Advanced or metastatic cancer survivors	4	4.3
Caregivers included	9	9.7
Older adult survivors	6	6.5
Providers	1	1.1
Racial/ethnic minority survivors	9	9.7
Long-term survivors (5 or more years since diagnosis)	5	5.4
Adolescent and young adult survivors	7	7.5
Hematopoietic stem cell transplant survivors	2	2.2
*Study design*		
Observational	47	50.5
Cohort (retrospective/prospective)^a^	43	91.5
Prospective component^b^	33	76.7
Cross-sectional^a^	3	6.4
Other^a^	1	2.1
Intervention^c^	46	49.5
Randomized controlled trial^d^	34	73.9
Non-randomized controlled trial^d^	3	6.5
Single arm^d^	8	17.4
Pilot^d^	18	39.1
*Primary focus*		
Acute psychosocial or physiological toxicity	25	26.9
Care delivery-care coordination	9	9.7
Health Promotion/Lifestyle	4	4.3
Late- or long-term effects	50	53.7
Methods	5	5.4
*Outcomes examined*^c^		
Biomarkers/biologic data	17	18.3
Physiologic	58	62.4
Psychosocial	19	20.4
Health behaviors/health promotion	5	5.4
Health-related quality of life	17	18.3
Healthcare utilization	14	15.1
Employment/financial outcomes	1	1.1
Mortality or survival, progression	10	10.8
Models or measure development	13	14.0
Other (includes feasibility and adherence outcomes)	14	15.0

^a^ percentage calculated with the total number of observational studies as the denominator

^b^ percentage calculated with the number of cohort studies as the denominator; prospective component is defined as a grant comprised of a single observational cohort study that is prospective or multiple observational cohort studies of which one is prospective and the other is retrospective

^c^ characteristic was coded as ‘select all that apply’, thus, percentage for characteristic categories will not add up to 100%

^d^ percentage calculated with the total number of intervention studies as the denominator

About half of the grants were observational studies (50.5%), of which the majority were of cohort study design (91.5%) (Table 2). Of the intervention studies, 73.9% were randomized controlled trials; 39.1% were pilot studies. The percentages of observational versus interventional grants differed by cancer type (Fig. 2), although the four most represented rare cancers in the portfolio had, generally, equivalent percentages of grants that were observational and interventional.

The primary focus of grants in the portfolio was most often late- or long-term effects (53.7%), with 26.9% focused on acute toxicities (i.e., physiological or psychological side effects or symptoms occurring within the first 6 months of diagnosis or treatment) and 9.7% focused on care delivery or care coordination (Table 2). A wide breadth of outcomes was examined in the portfolio, including physiologic effects (62.4%), psychosocial outcomes (20.4%), quality of life (18.3%) and healthcare related outcomes (15.1%). Approximately 18% included a biomarker outcome.

### Rare cancer portfolio in the context of SEER statistics

**Fig. 3:**
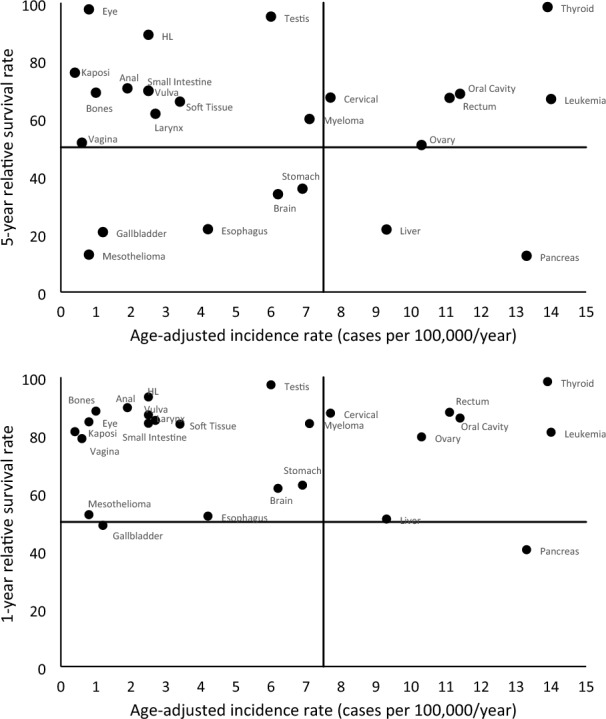
Rare cancer types by 2020 SEER age-adjusted incidence rates and 5-year and 1-year relative survival rates

Based on SEER statistics through 2020, 25 cancer types had age-adjusted incidence rates < 15 cases per 100,000 individuals in the US population (Supplementary Table 1). The 1-year relative survival rates of these cancers ranged from 40.5% (pancreas) to 98.5%; (thyroid); the 5-year relative survival rates of these cancers ranged from 12.5% (pancreas) to 98.5% (thyroid) (Supplementary Table 1). The majority of rare cancer grants in the portfolio (52.7%) were focused on the higher incidence/higher 5-year survival cancer types (Fig. 3), which included leukemia, ovarian, oral cavity, and cervical cancers. Although included among the higher incidence/higher 5-year survival cancer types, thyroid and rectal cancer were the focus of only one grant in the portfolio each. The lower incidence/higher 5-year survival cancer types were the focus of 11.8% of the grants, of which only testicular cancer, osteosarcoma, sarcoma (soft tissue), and multiple myeloma were represented. No grants focused on Hodgkin lymphoma, cancer of the small intestine, anus, anal canal, anorectum, vulva, larynx, or any of the cancers with an incidence rate less than 1 (vagina, Kaposi sarcoma, or eye). The higher incidence/lower 5-year survival cancer types included liver and pancreas sites. There were only two grants in this incidence/5-year survival category, both of which were focused on the pancreas (2.2%); no grants focused on the liver. The lower incidence/lower 5-year survival cancer types included stomach, brain, esophagus, and gallbladder, and were the focus of 18 grants (19.4%); all 18 of these grants focused on brain cancer.

## Discussion

In the United States (US), for nearly all rare cancers, the 1-year relative survival rate is now at least 50%, with trends, in general, over the past 10 years showing slow but steady improvements for most cancer types [8]. With survival improving for many of these rare cancers, it is critical that survivorship needs for those diagnosed with these cancers be identified and addressed. The purpose of this report was to examine the NIH grant portfolio from FY2017 to FY2023 focused on survivorship among individuals diagnosed with rare cancers and to identify gaps in this area of science.

Overall, the number of grants focused on rare cancers has increased over the past seven years, as has the percentage of grants in the total survivorship portfolio that are focused on rare cancers. While these statistics are encouraging, approximately 85% of the rare cancer grants funded over this time period have investigated only one of four cancer types (or type categories)—leukemia, head & neck, ovarian and brain; few grants have focused on other rare cancer types. Notable gaps in the NIH grant portfolio include several cancers with high (> 50%) relative 1- and 5-year survival rates and incidence rates between 7.5 and 15 cases per 100,000—specifically, only five grants were funded from FY2017 to FY2023 focused multiple myeloma, three on testicular cancer, one on rectal cancer and thyroid, and zero on cervical cancer. Several of these cancers, as well as others that are not represented in the rare cancer survivorship portfolio, such as liver and stomach cancer, have higher incidence rates in certain minoritized populations [7], underscoring the fact that a lack of survivorship research and evidence-based standards of care may further compound health disparities in outcomes. Survivors of each of these cancer types are likely to undergo surgery, chemotherapy, radiation, and/or other treatments with adverse physiologic, psychological, social, and financial effects that potentially last years after their diagnosis. Yet, our results indicate that there is a paucity of research being funded by NIH examining survivorship issues or interventions in these, as well as other, rare cancer types.

Another notable finding of this analysis is that only 10% of grants were coded as having a primary focus on care delivery or care coordination. While collecting information on acute toxicities and late-effects, as well as examining effective ways to prevent, alleviate, or ameliorate these effects, is important and was the primary focus of 80% of the grants in the portfolio, determining the best ways to deliver and coordinate survivorship care for individuals diagnosed with rare cancers is vital. Current survivorship care guidelines have been developed primarily for common cancers, including breast and colorectal cancers [9, 10]. However, these guidelines do not necessarily apply to survivors of other types of cancer, especially those that are rare and for which less is known about the survivorship trajectory. In addition, given that primary care clinicians often provide components of survivorship care, including managing late- and long-term physical and psychosocial symptoms and comorbid conditions, the lack of evidence-based guidelines and models of care for these rare cancers can lead to unmet needs and poor health outcomes. More research on care coordination models tailored for rare cancer survivors is needed.

Only approximately 10% of the grants in the portfolio analysis included caregivers, a percentage that is similar to the percentage that was reported in the broader cancer survivorship portfolio analysis [6]. Caregivers of those diagnosed with a rare cancer may face additional burdens given the lack of information, resources, advocacy organizations, and support systems [11]. This may be especially true for family caregivers who have been shown to be particularly vulnerable to stress and need or seek support [12, 13]. Research is needed to understand the physical, mental, financial, and other challenges faced by individuals caring for those with rare cancers and how to best provide support.

One special population of interest that comprises about one-third of the total rare cancer grant portfolio is the pediatric cancer survivor population. In the US, it was estimated that 9,620 children (ages 0 to 14) and 5,290 adolescents (ages 15 to 19 years) would be diagnosed with cancer in 2024; rare cancer types comprise a greater proportion of the cancers diagnosed among children compared to adults [14]. Advances in treatment and supportive care over the past several decades have led to growing populations of pediatric and AYA cancer survivors [15, 16], the majority of whom will live well into adulthood but suffer from adverse physical, psychosocial, or behavioral outcomes after the completion of treatment [17, 18]. There has been a long-standing interest in the health of childhood cancer survivors in the US; for example, in 2018, the Childhood Cancer Survivorship, Treatment, Access, and Research (STAR) Act [19] was signed into law, and reauthorized in 2023, leading to allocated funding for grants focused on pediatric and AYA cancer survivor populations. Continued focus on pediatric and AYA cancer survivors diagnosed with both common and rare cancers will continue to help make progress in improving morbidity and mortality rates in this population, many of whom live for many years after their diagnosis.

About 25% of the grants identified for inclusion in this analysis were funded through the R21 mechanism, which is higher than the 18% reported for the overall survivorship portfolio [6]. The R21 mechanism is utilized to fund highly innovative projects that are in early and conceptual stages of project development [20]; R21 awards allow for short duration funding of two years. This finding suggests that a substantial portion of the NIH-funded research focusing on rare cancers is in the early development stage—perhaps either lagging behind research being conducted for common cancers and/or not progressing to larger research project grants or cooperative agreements (e.g. R01, R37, U01). Results of this analysis also showed that 41% of the rare cancer grants were MPI grants, compared to 35.4% of the overall survivorship portfolio [6]. The higher percentage of MPI grants may reflect the complex nature of the rare cancer grants and an indication that, because these cancer types are understudied, there is a need for multidisciplinary and experienced teams with access to adequate resources to carry out projects.

Conducting survivorship research on rare cancers is challenging, primarily because there are fewer cases diagnosed each year compared with common cancers. It may be difficult for single institutions to recruit enough cases to achieve adequate statistical power for a study, necessitating multiple sites for recruitment, which can be expensive, or use of an existing larger resource. One such type of resource is a cancer consortium. Consortia have been developed for several rare cancer types—most notably brain and ovarian cancer, cancer types which are well-represented in the current NIH rare cancer grant portfolio—and could serve as models for other rare cancer types. It is also critical that innovative methods and strategies be developed to tackle small sample size issues that researchers involved in rare cancer research face.

Several limitations of this analysis should be considered. First, only studies that focused exclusively on a rare cancer type or types were included. Thus, if a grant in the larger survivorship portfolio included a rare cancer but was not specifically focused on the rare cancer (e.g., a common cancer was also studied), the grant was excluded. This may have led to an underestimation of the number of grants that are generating knowledge on rare cancer survivorship; although the excluded grants most often included a common cancer with the rare cancer because they did not have sufficient statistical power to examine the rare cancer alone. Second, this analysis did not include grants funded by other agencies or foundations. It is acknowledged that rare cancer research is being funded by other entities, including the Department of Defense, which has a robust rare cancer grant program. Yet, even within these other rare cancer programs, survivorship has not been a focus.

In 2016, the Cancer Moonshot was launched by the US Government to speed progress in the fight against cancer. In February 2022, the Cancer Moonshot was reignited, and new goals were set to reduce age-standardized cancer mortality rates by at least 50% over the next 25 years and to improve the experience of people living with and surviving cancer and their families [21]. Following this relaunch, NCI released, in April 2023, a new National Cancer Plan to serve as roadmap to achieving these goals [22]. This plan established eight major goals, several of which relate to empowering research in rare cancers, including eliminating health inequities and delivering optimal care. To reach the goals of both the new National Cancer Plan as well as the relaunched 2022 Cancer Moonshot, progress for all cancers, not just those that are most common, is critical. The importance of addressing rare cancers is also a focus of the European Union (EU), as evidenced by the existence of the EU Rare Cancer Working Group and development of the EU Rare Cancer Agenda 2030 [23]. Conducting research pertaining not only to the prevention, detection, and treatment of rare cancers, but also to the identification of survivorship needs of individuals diagnosed with rare cancers and the development of interventions to address these needs, is critical to reducing mortality rates from cancer and improving the health of all cancer survivors around the world.

## Supplementary Information

Below is the link to the electronic supplementary material.

Supplementary file1 (XLSX 5506 KB)

## Data Availability

The list of grant numbers included in this portfolio analysis are provided in Supplemental Table 2. Surveillance, Epidemiology, and End Results (SEER) Program data utilized in this manuscript are publicly available at https://seer.cancer.gov.
